# Study of the biological effectiveness of a nanosilver-epidermal growth factor sustained-release carrier

**DOI:** 10.3892/etm.2013.952

**Published:** 2013-02-06

**Authors:** JIAN-DA ZHOU, SHAO-HUA WANG, RUI LIU, CHUN-JIAO ZHOU, KE CAO, JIN-YAN LIU, YAO CHEN, FENG-HUA CHEN

**Affiliations:** 1 Third Xiang-Ya Hospital, Central South University, Changsha 410013, P.R. China; 2 College of Science, Hunan Agricultural University, Changsha 410128, P.R. China; 3 Xiang-Ya Hospital, Central South University, Changsha 410008, P.R. China

**Keywords:** epidermal growth factor, nanosilver, antibacterial, wound, cell proliferation

## Abstract

The aim of the present study was to elucidate the biological effectiveness and character of a nanosilver-epidermal growth factor (EGF) sustained-release carrier. This was synthesized using the self-assembly method and then characterized by transmission electron microscopy and UV spectrophotometry. The biological activity of the sustained release carrier was determined through cytological, bacteriological and wound-healing experiments. The results showed that the nanosilver-EGF sustained-release carrier was well dispersed with uniform particle size and that it had good antibacterial properties similar to those of nanosilver. The nanosilver-EGF sustained-release carrier is superior to EGFs in effectively promoting cell division and proliferation. The results of the wound-healing experiments provide evidence of its curative effects.

## Introduction

Nanoparticles have been increasingly applied in biomedical, pharmaceutical and clinical medicine. Among them, nanosilver is widely used in clinical burns and dental and urological practices (1–3). In cytotoxicity and animal experiments, many studies demonstrated that nanosilver had no toxicity, but had a high antibacterial activity (4–6). Tian *et al* (7) identified that local use of a nanosilver wound dressing not only accelerated healing, but also improved the appearance of scars. Madhumathi *et al* (8) and Ong *et al* (9) showed that a nanosilver/chitosan dressing effectively resists *Staphylococcus*, colonic and other bacteria and shows good hemostatic effects in the treatment of burn wounds.

Growth factors are a class of peptides or proteins that are able to regulate cell growth and differentiation and promote tissue healing (10). Epidermal growth factors (EGFs) have achieved good clinical results (11), but their *in vivo* stability is poor; they are vulnerable to degeneration or inactivation and are easily diminished in the blood circulation. Development of a stable, safe and effective preparation has become a challenging and practical focus for pharmacological research (12). Existing studies have demonstrated the role of controlled- or sustained-release formulations that are prepared with nanoparticles as a carrier. Carriers effectively protect drugs against inactivation and achieve sustained, controlled or even targeted release, thus significantly improving the curative efficacy and reducing toxic side-effects (13).

Anti-inflammatory nanosilver combines with EGF to create a sustained-release carrier that has resistance to infection and sustained-release properties similar to those of EGF. The resultant carrier is able to promote the repair of skin damage and compensate for the wound infection-induced low activity when EGF is used alone. Our research group has preliminarily determined the optimal particle size and complex conditions for nanosilver and EGF. In the present study, a sustained-release carrier was prepared using a 20-nm nanosilver particle and EGF using the self-assembly method (14) to elucidate the biological effects and character of the carrier.

## Materials and methods

### 

#### Preparation and characterization of the silver nanoparticle-EGF sustained-release carrier (NanoAg-EGF) solution

A 5,000-ppm solution of silver nanoparticles (5 ml) was magnetically stirred with 50 *μ*g/ml EGF solution (10 ml) and adjusted with HCl-Tris buffer to pH 7.0. The volume was set at 50 ml and scattered for 30 min with an ultrasonic dispersion machine. The solution was then placed in a 37°C water bath overnight for 12 h to obtain a 500-ppm solution of the sustained-release carrier (final concentration of silver nanoparticles, 500 ppm; final concentration of EGF, 10 *μ*g/ml). The sustained-release solution (25 ppm) was similarly prepared (final concentration of silver nanoparticles, 25 ppm; final concentration of EGF, 10 *μ*g/ml). Freeze-dried EGF powder (1 mg) was dissolved in sterile distilled water to prepare the EGF-alone (10 *μ*g/ml) and a 500-ppm solution of silver nanoparticles (1 ml) was set at 10 ml to obtain 500-ppm solution of silver nanoparticles and was set at 50 ml to obtain 100-ppm solution of silver nanoparticles. All solutions were stored in a sterile bottle in a refrigerator at 4°C for further use.

#### Transmission electron microscopy

The 500-ppm nanosilver particle solution and the 500-ppm NanoAg-EGF solution were observed with transmission electron microscopy.

#### Ultraviolet visible (UV-VIS) spectrophotometry

The 25-ppm sustained-release carrier solution was centrifuged at 20,000 rpm for 2 h and the supernatant was then collected for detection. Double distilled water served as a reference sample adjusted to ‘A0.000’. The test sample was placed into the cuvette followed by the ordered measurement of the EGF-alone (10 *μ*g/ml), NanoAg-alone (100 ppm), NanoAg-EGF (25 ppm) and sustained-release supernatant groups.

#### Cell proliferation experiments with the NanoAg-EGF

The KMST6 skin fibroblast cell line was resuscitated and added to a culture medium to obtain a triturated cell suspension. The cell suspension was subpassaged at 5×10^5^/ml and cultured in a 75-ml culture flask at 37°C in a 5% CO_2_ incubator until passages 4–8. The suspension was then seeded onto a 96-well cell culture plate with 100-*μ*l/well at 37°C in a saturated humidity of 5% CO_2_ for 24 h. The culture medium was discarded and the sample was added to the EGF (10 mg/l), nanosilver solution (500 ppm), nanosilver-EGF (500 ppm) group, nanosilver-EGF combination group (NanoAg+EGF) and control groups. Each group had eight double wells; there were four repeated plates in each group with 100 *μ*l of solution in each well. All samples were cultured at 37°C in a 5% CO_2_ incubator. One culture plate was taken out at 12, 24, 36 and 48 h for an MTT colorimetric test. The cell growth curve was then plotted.

#### Antibacterial tests with the NanoAg-EGF

Five pathogenic microorganisms, namely *Staphylococcus aureus* (ATCC 29213), *Escherichia coli* (ATCC 25922), *Pseudomonas aeruginosa* (10102), *Candida albicans* and *Streptococcus pneumoniae*, were incubated with the culture medium (including the nutrient broth and agarose media) at 4°C. Each bacterial species was repeatedly incubated on five plates; the concentration of bacterial suspension was estimated turbidimetrically and inoculated onto petri dishes at concentrations of 5×10^5^ to 5×10^6^ cfu/ml. The bacterial suspension was smeared onto the surface of a nutrient agar plate and the petri dishes were dried at room temperature. The sterile, dried filter paper (5-mm diameter) was collected and added to 5 *μ*l of the reagents to prepare the antibacterial slices. The samples were cultured for 24 h in a 37°C incubator. The diameter of the antibacterial ring was measured with compasses and a caliper and the measurements were repeated three times.

#### Wound healing experiments with the NanoAg-EGF

A wound was made on each side of the spinal cord in 15 rats, which were intramuscularly injected with 5 mg/kg gentamicin (equivalent to the plasma concentration in human adults) once daily for 3 days for the systemic anti-infective treatment. The wounds were randomly assigned to the NanoAg-EGF, NanoAg-alone, EGF-alone, NanoAg+EGF or normal saline control groups. Each wound was administered its assigned treatment once daily. The drugs infiltrated the whole wound by sterile syringe infusion and each wound received 0.25 ml of drug per treatment. Images of the wounds were captured on days 3, 7 and 12 and at the time of wound healing. The non-healing area was calculated using a computer image analysis system (CAD software). The healing rate was calculated as follows: Healing rate = (initial wound area − nonhealing area) / initial wound area × 100.

#### Statistical analysis

Measurement data are expressed as mean ± standard deviation and were analyzed using SPSS 13.0 software. Differences among the groups were compared using an analysis of variance. Pairwise comparison was performed with the LSD test. P<0.05 was considered to indicate a statistically significant result.

## Results and Discussion

### 

#### Characterization of the NanoAg-EGF with transmission electron microscopy

**Figure 1:**
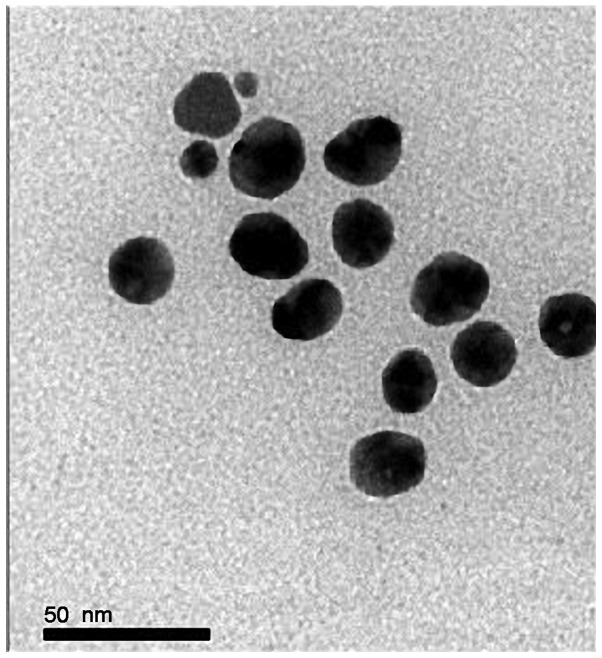
Transmission electron microscopic image of the silver nanoparticles (magnification, ×500,000).

**Figure 2:**
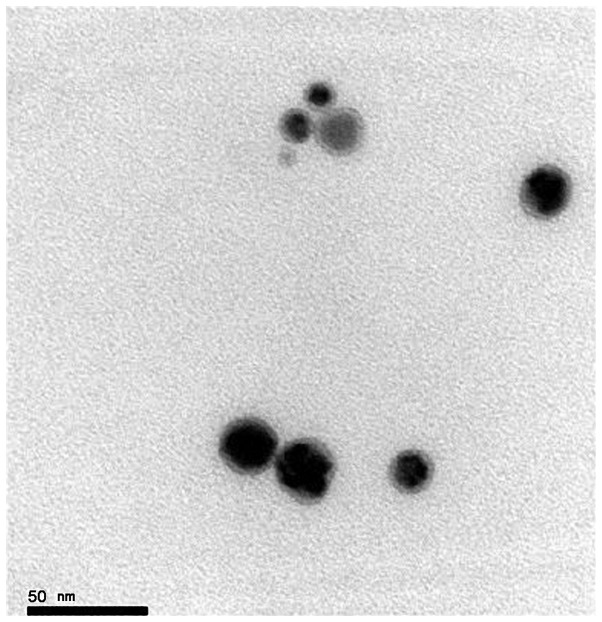
Transmission electron microscopic image of the nanosilver-EGF complex (×500,000). EGF, epidermal growth factor.

A transmission electron microscopic image of nanosilver at 500 ppm is shown in Fig. 1. The characterization analysis showed that the silver nanoparticles were spherical with uniform distribution, showing no agglomeration or growth, and with a particle size of 15–25 nm. A transmission electron microscopic image of the NanoAg-EGF is shown in Fig. 2. Lightly stained EGF covered the surface of the spherical silver nanoparticles and formed a nebula-like shadow, which was surrounded by silver nanoparticles. This is objective evidence of EGF adhesion on the silver nanoparticles.

Peptides and proteins are increasingly being used in clinical practice, and the preparation method using nanoparticles has been developed (15) so that they may serve as carriers of these peptide and protein drugs. The use of biodegradable polymers or inorganic nanoparticles as carriers of peptides and proteins, thus achieving a sustained-release effect, is a current research focus (16–18).

#### UV-VIS characterization

**Figure 3:**
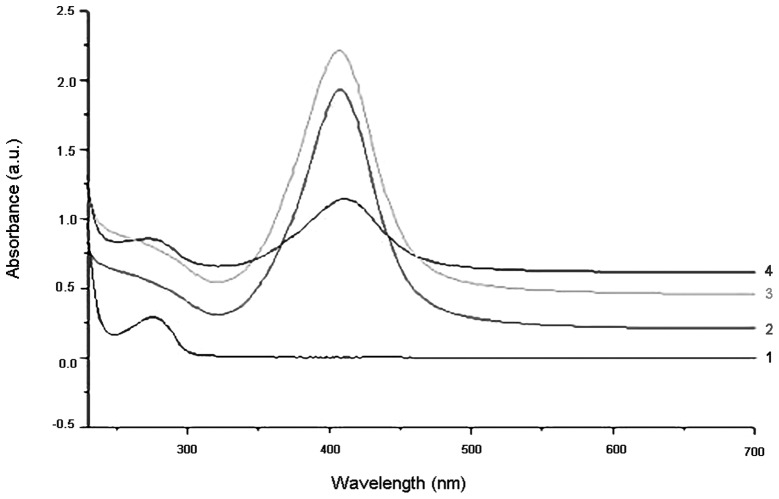
Ultraviolet absorption spectrum of nanosilver-EGF complex. (1) Absorption spectrum of EGF. (2, 3) Absorption spectrum of nanosilver-alone group (100 and 500 ppm, respectively). (4) Absorption spectrum of nanosilver-EGF sustained-release group (25 ppm). EGF, epidermal growth factor.

The UV-VIS absorption spectra of the NanoAg-alone, EGF-alone and NanoAg-EGF solutions are shown in Fig. 3. The first absorption peak in curve 4 was exactly the same as that in curve 1, indicating that free EGF was present in the NanoAg-EGF solution. The second absorption peak of curve 4 shifted to the right compared with the absorption peak in curves 2 and 3, with the UV-VIS absorption peak of the NanoAg-EGF 5 nm away from that of the silver nanoparticles. This evidence suggests that the EGF acting with the nanosilver in the NanoAg-EGF solution produced a nanosilver-EGF complex.

According to the Mie theory (19,20), the plasma absorption peak gradually red-shifts with increasing nanoparticle size. When the size of the silver nanoparticles increased, the plasma absorption peak red-shifted, which is strong evidence for adhesion of nanosilver to EGF. EGF effectively adsorbed to the surface of the silver nanoparticles, indicating that the NanoAg-EGF was successfully prepared. This is consistent with a previously described outcome (21) showing that nanosilver are able to act with proteins, resulting in the alteration of their spectrum.

### NanoAg-EGF promotes cell proliferation

#### Growth of fibroblast cell culture

**Figure 4:**
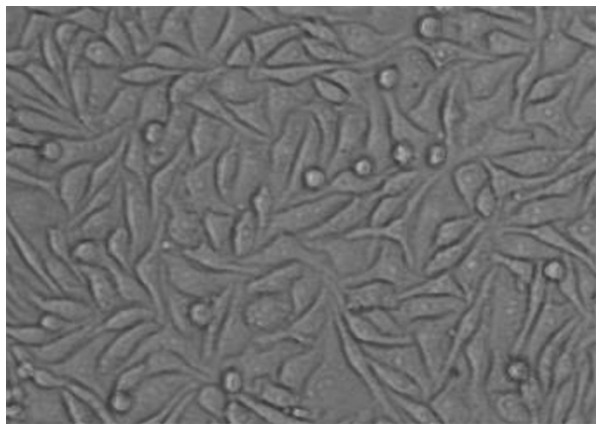
Light microscopic image of human dermal fibroblasts at 36 h subsequent to hematoxylin-eosin staining in the nanosilver-EGF complex group. EGF, epidermal growth factor.

Under light microscopy, the number of dermal fibroblasts was increased, the distribution was dense in the whole field of vision and the cells were mostly spindle-shaped. Hematoxylin-eosin staining showed a pink-stained cytoplasm and blue-stained nuclei. The cells were fusiform-shaped with several processes or star-shaped and flat. The outlines were clear and the nuclei were oval. There were no significant differences in the morphology of the treated cells (Fig. 4).

**Figure 5:**
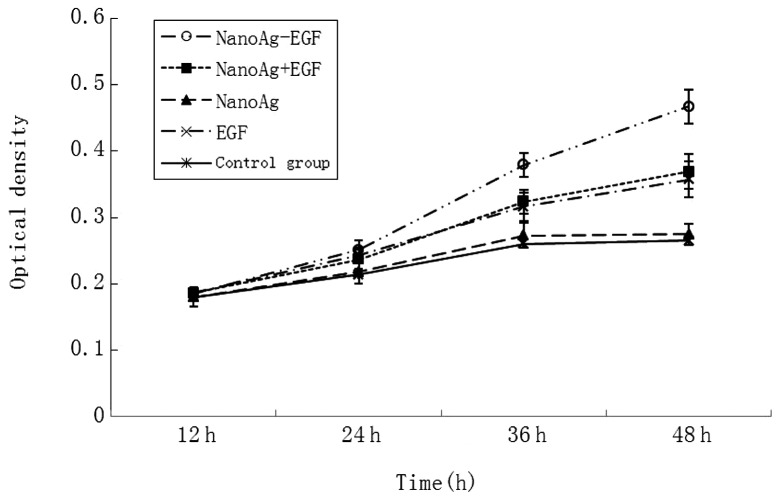
Proliferation of human dermal fibroblasts in each group at 12, 24, 36 and 48 h. EGF, epidermal growth factor; NanoAg-EGF, nanosilver-epidermal growth factor sustained-release carrier; NanoAg+EGF, nanosilver-EGF combination; NanoAg, nanosilver-alone.

The absorbance value in each group was detected at 12, 24, 36 and 48 h (Fig. 5). There was no significant difference in the absorbance value of the human fibroblasts at 12 h (0.180±0.011 vs. 0.186±0.009; P>0.05). Cell proliferation was apparent as time went by. Cell proliferation in the groups containing EGF (i.e., the NanoAg-EGF, NanoAg+EGF and EGF-alone groups) was significantly evident compared with that in the nanosilver and control groups (P<0.05). At 36 and 48 h, cell proliferation in the sustained-release carrier group was the most evident. The absorbance values were 0.359±0.027 and 0.467±0.026, respectively, which were significantly greater than those in the combined (0.324±0.022 and 0.359±0.027) and EGF groups (0.316±0.019 and 0.357±0.016; P<0.05). This is evidence that cell proliferation was faster and more stable following 24h in the sustained-release carrier group. Accordingly, it is speculated that the NanoAg-EGF is able to greatly promote cell proliferation and that this ability is closely attributed to the sustained-release effect of the silver nanoparticles on EGF.

In the experiments of the present study, the cell proliferation was promoted to varying degrees in the groups, but no significant difference was observed. This may be as the biological effects on the promotion of cell proliferation remained in the initial phase within a short duration of EGF action. Cell protein, DNA and RNA synthesis was significantly increased, but no quantitative change in cell number was identified. When fibroblasts were treated with EGF, the cell cycle duration was ∼10 h and DNA synthesis started at 8 h and became active. Subsequent to 24 h of cell culture, the number of cells increased in the NanoAg-EGF, EGF-alone and NanoAg+EGF groups with significant differences compared with the NanoAg-alone and control groups. This provides evidence that EGF promoted cell proliferation. Subsequent to 36 h of cell culture, the number of cells in the NanoAg-EGF group was significantly higher than that of the EGF-alone and NanoAg+EGF groups, suggesting that the concentration of sustained-release carrier was better than that of the EGF-alone and explaining the cell proliferation at 36 and 48 h.

#### Antibacterial test of NanoAg-EGF

**Figure 6:**
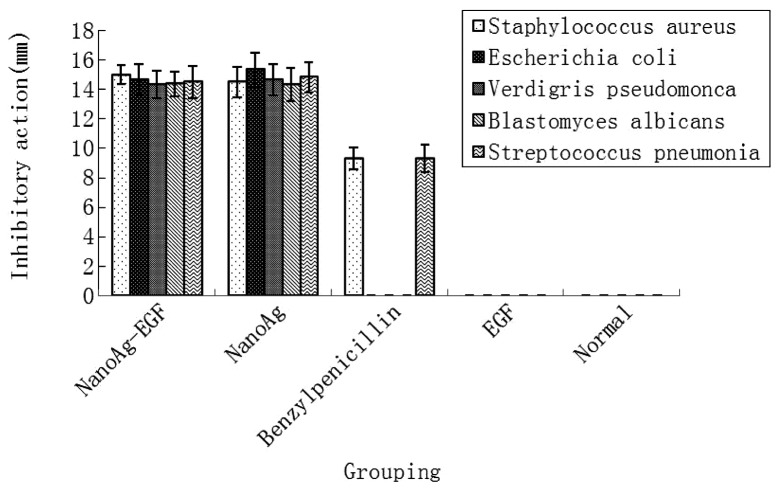
Inhibitory action of the experimental group against five pathogenic microorganisms. (1) NanoAg-EGF group. (2) NanoAg group. (3) Benzylpenicillin positive control group. (4) EGF group. (5) Normal control group. EGF, epidermal growth factor; NanoAg-EGF, nanosilver-epidermal growth factor sustained-release carrier; NanoAg, nanosilver-alone.

The inhibitory action of each treatment group was compared in five pathogenic microorganisms. As shown in Fig. 6, the NanoAg-EGF and NanoAg-alone groups showed good antibacterial properties against five pathogenic microorganisms, namely *Staphylococcus aureus*, *Escherichia coli*, *Verdigris pseudomonas*, *Blastomyces albicans* and *Streptococcus pneumoniae*, with no statistically significant difference in antimicrobial resistance between the two groups (P>0.05). In the positive-control benzylpenicillin group, weak antibacterial activity occurred only against *Staphylococcus aureus* and *Streptococcus pneumoniae* and was significantly lower than that in the NanoAg-EGF and NanoAg-alone groups (P<0.05). In the normal saline control and EGF groups, there was no antibacterial effect on the five pathogenic microorganisms.

The antibacterial effect of the NanoAg-EGF was determined. The NanoAg-EGF and nanosilver groups showed strong inhibitory actions against the five pathogenic organisms, while the EGF-alone and normal saline control groups showed no inhibitory effects. In the positive-control group, benzylpenicillin sodium was only resistant to *Staphylococcus aureus* and the antibacterial effect was significantly lower than that of the NanoAg-alone and NanoAg-EGF groups. There was no significant difference between these two groups. The present experiments not only validate the antibacterial effect of nanosilver, but also confirm that nanosilver has a good inhibitory effect on *Staphylococcus aureus* and *Pseudomonas aeruginosa*, which readily demonstrate drug resistance.

### NanoAg-EGF promotes wound healing

#### Morphological observations

The wounds in the rats of the NanoAg-EGF group were cleaner than those in the other groups, with less leakage. The rats also had a mental status that was close to that of normal rats, a normal diet, vigorous activity and no hair loss. Wound healing in the other groups was relatively poor or even difficult, with more secretions and surrounding swelling, leading to formation of chronic ulcers.

#### NanoAg-EGF promotes wound healing in animal experiments

**Figure 7:**
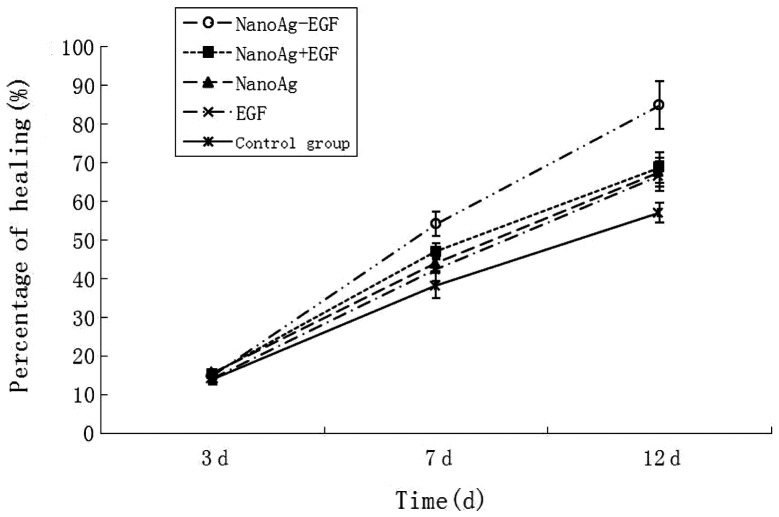
Comparison of the wound-healing rate in each group at 3, 7 and 12 days post-trauma. EGF, epidermal growth factor; NanoAg-EGF, nanosilver-epidermal growth factor sustained-release carrier; NanoAg+EGF, nanosilver-EGF combination; NanoAg, nanosilver-alone.

The statistical data on the wound-healing rate in each group are shown in Fig. 7. The wound-healing rate at 3 days post-treatment in each group ranged from 14.105±1.098 to 15.814±1.518% with no significant difference between the groups (P>0.05) at 7 and 12 days. The healing rates in the NanoAg-EGF group were 54.19±3.1137 and 84.933±6.147%, respectively, which were significantly higher than those in the other four groups (P<0.05). The wound-healing duration in the NanoAg-EGF group was 14.75±1.603 days, which was significantly shorter than that of the other four groups (NanoAg+EGF group, 17.25±1.422 days; EGF-alone group, 20.167±1.697 days; NanoAg-alone group, 17.083±1.505 days; and control group, 20.333±1.303 days; P<0.05). Thus, the wound-healing rate and duration were the highest and shortest, respectively, in the NanoAg-EGF group.

Numerous measures are used to improve wound-healing duration and quality, including infection control, active removal of necrotic tissue, correction of metabolism and application of exogenous growth factors. Wound healing is a key problem in plastic surgery and related research, and therefore the issue of how to speed up wound healing is evident in clinical research (22). In the present study, there was no significant difference in the wound-healing rate of each group at 3 days post-surgery, indicating that EGF was ineffective in the promotion of wound healing in the early inflammatory stage. Even if anti-infection measures are performed in a timely manner, wound edema and acute infection occur at 2 to 3 days post-trauma. Due to the marked change in the surrounding environment, cells on the wound surface remain in the shock stage and growth factors are not available. In addition, a certain amount of time is required to upregulate the exogenous EGF receptor, thus, no promotion of wound healing was identified in the NanoAg-EGF group at 3 days in the present study. The healing rate reached a peak in the NanoAg-EGF group 7 days subsequent to the injury with significant differences when compared with the other groups; the differences were most significant with time. The wound-healing duration in the NanoAg-EGF group was 4 to 5 days shorter than in the saline group and 2 to 3 days shorter than in the combination group. Although the combination group showed a better ability to promote wound healing at all time-points, there was no significant difference compared with the EGF-alone and NanoAg-alone groups. Therefore, it is speculated that combining the silver nanoparticles with EGF is not able to lead to a qualitative change in wound healing. In the NanoAg-EGF group, the healing rate was significantly higher than that of the other groups at 7 days, suggesting that the new formulations are able to avoid wound hydrolysis, induce a sustained and steady release of EGF and protect factors from wound hydrolysis and bacterial destruction prior to the adherent growth factor detaching from the silver nanoparticles. When the amount of growth factors on the wound surface decreases, the adherent growth factor gradually becomes free from the nanosilver and then binds with receptors that are able to repair cells and promote cell proliferation. Therefore, the wounds maintain a relatively high concentration of growth factors and wound healing is accelerated.

In conclusion, the experimental findings of the present study confirm that the described NanoAg-EGF solution is able to disperse well, that EGF adheres to the surface of silver nanoparticles and that growth factor activity and antimicrobial resistance coexist and effectively promote wound healing. Further studies are required to conclusively determine the clinical application and significance of these results.
